# Environmental Health Practice: An Identity in Crisis

**DOI:** 10.3390/ijerph23010090

**Published:** 2026-01-09

**Authors:** Jason Barnes

**Affiliations:** School of Psychology and Public Health, La Trobe University, Bundoora, VIC 3083, Australia; jason.barnes@latrobe.edu.au; Tel.: +61-3-9479-3686

**Keywords:** environmental health, environmental health practice, environmental health officer, environmental health practitioner, public health practice, health occupations, identity, branding, professional role, health protection

## Abstract

Environmental health practice is facing an identity crisis. Not because our work lacks impact, but because our work lacks a coherent narrative. Environmental health practice needs a brand that speaks our truth and secures our place in the future of health protection. The brand of environmental health practice must be more than a label. It needs to be an asset that our industry can leverage to build identity, recognisability, credibility, relevance and a unique value proposition. A strong brand will allow us to express the unique impact of our role and thus, it needs to capture the most central component of our work that unmistakably differentiates it from anything else. A rapid review of the literature was performed using systematic methods to discern a coherent narrative capturing the essence of environmental health practice. Through application of a critical lens shaped through professional experience, a novel narrative was synthesized of the key defining component of our work. This narrative establishes the work of environmental health practitioners as a unique value proposition and forms the basis for a brand that we can fearlessly project and reverberate. It represents the makings for an unmistakable identity. The future of environmental health practice depends on environmental health practitioners establishing a brand by building awareness of the unique value of our work.

## 1. Introduction

Environmental health practice is branded in many ways: compliance assessment, risk assessment, enforcement, education. Yet, none of these descriptions fully capture the defining function of our work. We are facing an identity crisis, not because our work lacks impact, but because our work lacks a coherent narrative. Fundamentally, a brand is the definition of a company or a product [1]. Environmental health needs a brand that speaks our truth and secures our place in the future of health protection. Until we can capture the key defining component of our work, we are at great risk of being branded as ‘irrelevant’. We know the inherent value of our work, but presently we lack the capacity to express it.

### 1.1. Our Impact

Both within and outside our profession, there has been a great challenge in measuring the impact of environmental health practice. As an environmental health middle-manager, I too struggled with this, particularly in satisfying the needs of my executive overseers who cut their teeth in the finance domain, where everything has a price. However, our challenge in measuring impact may be the consequence of using a lens that is too narrow, rather than an inability to identify suitable metrics and devices for measuring ‘non-events’ [2]. When viewing in the micro, it is difficult to discern the impact of environmental health. When we take a macro position, viewing environmental health in the global context, the impact of environmental health practice is abundantly clear.

Regions of the world where environmental health practitioners do not form a primary component of a health protection system provide us with a clear point of comparison. The life expectancy of their children is brief, the threat of communicable disease is imminent, exposure to environmental health hazards is importunate and the lack of social and environmental justice is flagrant [3]. While the critics will be quick to remark that other health determinants, such as poverty, are the most probable cause for these public health outcomes, the advocacy and innovative work of environmental health practitioners would certainly contribute to enabling better public health outcomes [4]. When we reflect on it in these terms, the impact of environmental health practitioners on public health outcomes is profound. Thus, the question is not of the importance and value of the work we do, but in how we are best to express and progress it.

### 1.2. What Do We Need in a Brand?

The brand of environmental health practice needs to be more than a label. It needs to be an asset that our industry can leverage to build identity, recognisability, credibility, relevance, and a unique value proposition. A strong brand offers our industry immediate recognition by those who stand to benefit from our work, and to those with a problem that only our industry can solve [5]. Thus, in establishing our identity, we need our brand to offer tangible, interpretable and relatable representations of our work, but also a basis for differentiating our work and its implicit value from the work of others within the broader public health space [5,6]. A good brand will allow us to differentiate our role, our importance and our value and assert our place within the health protection domain, our sub-category of public health [6,7]. In doing so, our brand does not need to capture every aspect of the work that we do. Instead, it needs to capture the essence of our work, its most central component, and the aspect of our work that unmistakably differentiates it from anything else.

### 1.3. The Trouble with Our Inherited Brands

#### 1.3.1. Compliance Assessment and Enforcement

Compliance assessment and enforcement have been long-standing means of defining our work. It is certainly something that features in our work, and the earlier we are in our professional development, the more likely our work will align with these descriptions [8]. However, there are perils that come with the compliance and enforcement labels. Firstly, it foregoes the need for scientific knowledge that underpins our work, meaning we are rendered as simply observers [9]. Furthermore, it threatens our intellectual capital because an unskilled workforce is capable of undertaking compliance assessment [9]. Thirdly, it removes our capability to fix public health problems and address environmental health hazards, as it narrows the scope of the role to solely applying the law [10]. Additionally, it licenses our work to be measured using spurious metrics, such as measuring the number of inspections, the number of non-compliances, the number of enforcement actions, rather than measuring factors that reflect the true value of our work and our impact on public health. Finally, and perhaps most pertinently, is that compliance assessment comes with an additional label, categorising our work as ‘red tape’ [11]. Being categorised in this way makes environmental health practice extremely vulnerable to being diminished or swept away entirely when governments pursue neoliberalist ideals and act to ‘cut red tape’. Our work is invested in protecting public health, not regulatory encumbrance and thus is not adequately defined as a compliance assessment.

#### 1.3.2. Human Health Risk Assessment

This label offers a distinct improvement over compliance assessment because it recognises scientific methods and evidence to inform the decisions we make in protecting public health. It bases our work on sound principles provided by toxicology and broader scientific inquiry [12]. The trouble, however, is the positioning of the environmental health practitioner within the work. While we certainly assess risk to human health in almost every piece of work we do, particularly by comparing dose and exposure, or likelihood and consequence [13], it is only a part of the function we perform. This does not meet our needs of a brand because it does not holistically capture the central component of the work that we do and thus, the value our work delivers. Our work goes beyond human health risk assessment to analyse health issues within their context, examine the physical and social systems and structures to identify causative or facilitative aspects, and to place suitable interventions to protect public health.

#### 1.3.3. Inspection and Investigation

Inspections and investigations are key functions of our work. Much of our time as environmental health practitioners is spent undertaking inspections and investigations [14]. The trouble, however, with adopting such labels is that they neglect to recognise why we undertake inspections and investigations. As such, there is no distinction between our work and those that inspect and investigate outside the public health realm [15], resulting in the health protection impetus being lost. These are descriptions of the means of our inquiry, rather than the purpose of our inquiry [16], and thus, they promote an obdurate means to measuring our performance. Again, the number of inspections completed, the number of days taken to complete an investigation, and the number of violations identified are all measures directly related to this type of work, but certainly not relevant to their impact on public health [17].

### 1.4. The Rewards of an Unmistakable Identity

By capturing the key defining function of our work, it allows us to build evidence to inform how best we do it, it allows us to test and refine it to know that it is effective, it provides a basis for us to justify the great value of our work, it allows us to find aligned professions that we can collaborate with, and it allows us to design education and guidance for current and future environmental health practitioners.

This branding extends far beyond justifying our work; it is the first step in transforming our practice into one that is evidence-informed, capable of sustainability and recognisable for its impact. A brand identity allows for the translation of our work into a true value proposition to the public [18] and the protection of their health.

## 2. Materials and Methods

### What Is It That We Do Then?

Before forging a contemporary and ardent brand for environmental health practice, it is important to first establish a clear definition of what is being branded. The World Health Organization’s 1990 definition of environmental health offers broad insight into the nature of environmental health practice:

*“Environmental health comprises those aspects of human health and disease that are determined by factors in the environment. It also refers to the theory and practice of assessing and controlling factors in the environment that can potentially affect health”*

P.18, World Health Organization, Regional Office for Europe [19]

While this definition is valuable for its universality and resonance, its lack of specificity renders it a deficient foundation for the construction of an unmistakable identity.

In order to shape a clearer perspective in defining environmental health practice, a rapid review of the literature was conducted. The review was not aimed at providing a comprehensive account of environmental health practice, but instead at providing a pragmatic and functional basis from which a contemporary description of practice could be synthesized. A search protocol using search terms (“environmental health officer” OR “environmental health practitioner” AND “environmental health practice”) was deployed and these search terms were limited to title and/or abstract only. The search was limited to articles in academic journals, which were sought using the declared protocol from six online databases: ProQuest, Scopus, PubMed, Web of Science, CINAHL (EBSCO) and EMBASE in September 2025. The articles collected were then subjected to title and abstract screening and full text screening with the use of inclusion criteria ‘defines, describes or characterises environmental health practice’. Although a systematic procedure was followed, the screening was conducted by an individual, which meant the risk of bias could not be fully mitigated.

## 3. Results

**Figure 1:**
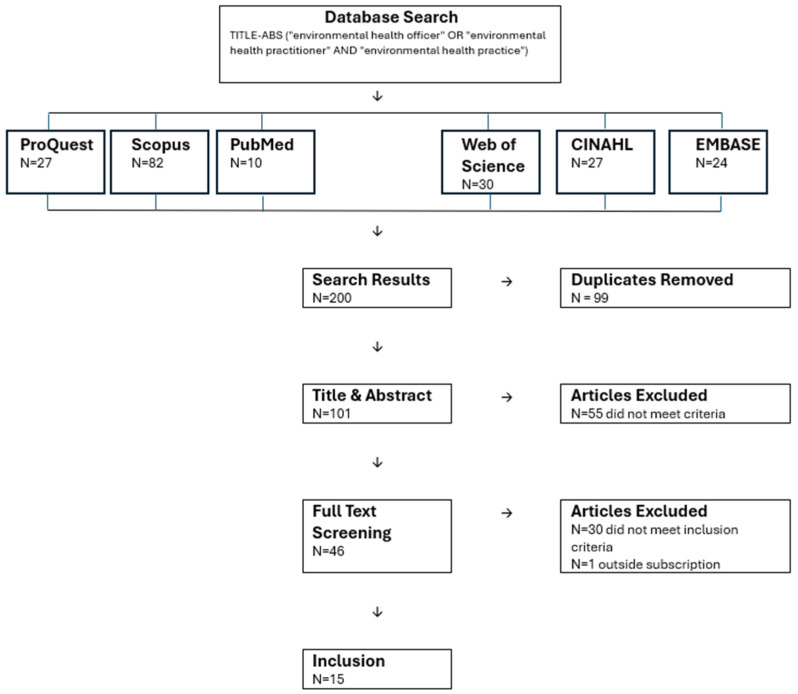
Summary of search results and screening procedures.

A summary of search results and screening procedures is shown in Figure 1.

Of the 200 results yielded from the search, 99 were removed as they were duplicates, a further 55 were removed through title and abstract screening, and a further 31 were excluded following full text screening. A total of 15 articles were found to define, describe or characterise environmental health practice, and these have been summarised in Table 1.

## 4. Discussion

### 4.1. Our Descriptions of Environmental Health Practice

Each of the 15 descriptions of environmental health practice, while offering unique perspectives, maintain clear alignment with the description advanced by the World Health Organization [19]. Collectively, these accounts recognise environmental health practice as fundamentally focused on the prevention of disease at the population level. Health protection consistently emerges as the central paradigm underpinning practice, though subtle variations exist in how this protective function is conceptualised and enacted.

Some descriptions portray protection as shielding individuals from harmful environmental exposures, while others adopt a broader lens, emphasising the safeguarding of the public from illness [29], or even prevention of deviation from a nominal state of complete health [21,31]. These nuanced distinctions reveal underlying tensions with regard to the settings in which environmental health practice occurs, the tools utilised, and the nature of interventions deployed by practitioners.

Accordingly, environmental health practice may be interpreted as involving direct environmental interventions to control hazards, or alternatively, as encompassing socio-behavioural strategies or even considering socio-ecological factors and social determinants of health in preventing harmful exposures. Across the chronology of these descriptions, there is a recurring notion to expand the traditional scope of environmental health practice from a narrow health protection paradigm toward a more holistic health promotion approach [23,24,27,28]. Notably, some of the more contemporary accounts explicitly incorporate social and psychosocial factors as environmental determinants of health [33].

When viewed through a temporal lens, an evolution is apparent within the descriptions where integration of risk assessment and risk management receives greater focus in later years [26,30,32,33]. Moreover, contemporary perspectives make a distinction between routine environmental health practice and practice conducted in the context of public health emergencies [29,32,33]. Thus, while the descriptions captured in the literature generally align on the basis of environmental health practice being a health protection function occurring at the population level, inconsistencies among the descriptions hinder the natural emergence of a coherent narrative that is essential to brand establishment. Instead, it is necessary to establish a description of the key function of our work by drawing on the literature, combined with reflection on experiences of environmental health practice.

### 4.2. Forging a Coherent Narrative

For environmental health practice, while the hazards at the centre of our focus are environmental and often insentient, and the data that we capture and interpret are both environmental and social, the interventions we apply are almost always within the social domain. We inform, educate, compel and punish duty holders, people (or corporations, comprising people) to protect public health; it is rare for environmental health practitioners to intervene directly in the environment, as is inferred by many of the descriptions of environmental health practice, particularly by those earlier descriptions [34].

As environmental health practitioners, our work focuses on ‘scientifically and systematically assessing the adequacy of how duty holders manage risk to public health from environmental health hazards’ and ‘as appropriate, applying interventions to motivate or improve the management of risk to better protect public health’.

We use empirical and social science to inform our assessment of risk and risk management adequacy, to devise suitable interventions, and to effect better protection of public health [34,35]. We team this scientific method of inquiry and reasoning with our regulatory powers to inspect and investigate, inform and educate, compel and enforce [34]. Our interventions are almost always within the social domain, requiring actions or changes in the practices of people. An illustration of how the narrative aligns with key domains of environmental health practice and emerging issues in the field is provided in Table 2.

### 4.3. A Name, a Narrative, a Brand, a Future

So, what does this all mean for a brand? Do we need to change our name to fit with our newfound narrative? Certainly, the corporate world provides endless examples of how a brand can be almost completely invested in a name [36]. Yet, the trouble we are attempting to overcome is not that of our name and its prominence over other competitors in the market, but rather the visibility and tangibility of who we are, what we do, and the impact we have. For now, environmental health practitioner remains relevant, and while the debates within our industry will continue about the best title for our role, if we manage to establish and embed our brand well enough, the quest for the most fitting title will prove irrelevant. If we manage to shape and share our brand, there will be no question of what an environmental health practitioner does and the value of their work.

### 4.4. The Path of Transition

How can we best share this narrative with the world? How do we begin building the brand of environmental health practice? The recent progress made by epidemiology in building a brand may hold some clues. Although the COVID-19 global pandemic brought great suffering and hardship to our global community, it provided a unique opportunity for the field of epidemiology to build its profile by meeting unmet community needs. The unprecedented nature of the global pandemic led to great uncertainty at all levels of society. It hampered decision-making, resulting in acute impasse for individuals [37], communities, organisations, policy makers, and governments by introducing profound levels of uncertainty [38]. The swathe of ambiguity created by the exceptional circumstances shrouded the best path of action, the next development, the state of the future, and thus, it invoked widespread uneasiness and discomfort. A need was created for answers that could ease the discomfort of our communities in the face of this uncertainty and to overcome the crippling impasse faced in decision-making; a market demand was created for the unique value of the work of epidemiology. Epidemiology became the trusted guide to help humanity navigate and forge a path through the pandemic. It became a vessel through which science could be translated and accessed by the public [39]. Hence, epidemiology quickly gained prominence, familiarity, exigency, voice and reach [40]. The identity of epidemiology and the unique value of its work developed into a brand that was immediately recognisable.

Although the COVID-19 pandemic presented unique circumstances conducive to brand development for epidemiology, a public health emergency is not pre-requisite for environmental health practice to do the same. Instead, the opportunity to showcase the unique value of our work is already at hand in the work we do every day. It is important for environmental health practitioners to leverage these interactions in our daily work to highlight the value and importance of environmental health practice. Drawing on Aaker’s five B’s of modern branding [41], Table 3 outlines strategies for strengthening the brand of environmental health practice.

Building the brand of environmental health practice will require a concerted effort, where practitioners work as individuals and as a collective to apply these strategies at local, national and global levels. This will require environmental health practitioners to actively seek opportunities to enhance both visibility and voice. We must step beyond the comfort of quiet competence to become confident advocates for the brand of environmental health practice. Our task is to clearly and consistently demonstrate to our communities that we are always present and always working to protect their health. This demands that we be assertive in articulating the value of our work in every interaction, in every conversation, in every budget bid, in every directive and in every publication.

## 5. Conclusions

Environmental health practice is experiencing an identity crisis. This is not because our work lacks impact, but because it lacks a coherent narrative to express its unique value. Without a strong and ardent brand, environmental health practice risks being branded as irrelevant. To secure its future in health protection, environmental health practice must establish and embrace a brand as a strategic asset that builds identity, credibility, relevance and recognizability. A rapid review of the literature was undertaken to synthesize a contemporary narrative to underpin this brand. Environmental health practice can be defined as ‘scientifically and systematically assessing the adequacy of how duty holders manage risks to public health from environmental health hazards’ and ‘as appropriate, applying interventions to motivate or improve the management of risk to better protect public health’. By projecting and reverberating this brand, environmental health practitioners can strengthen recognition of the unique value of their work and secure the profession’s place in the future of health protection.

## Figures and Tables

**Table 1:** Descriptions of environmental health practice.

Author	Type	Description of Environmental Health Practice
Bond, 1959 [20]	C	Described environmental health practice as applying skills and techniques to eliminate problems attributed to defects in the physical environment, controlling exposures to hazardous biological and chemical materials in the environment, and the skills and techniques detailed to include monitoring, sampling, epidemiological investigation and health education.
Morgan, 1975 [21]	C	Positioned environmental health practice as the first line of defence for humans against disease, describing it as a means of arresting the causative agents of disease before they reach the human body. Environmental health practice is described as maintenance of the environment as a means to alleviate health burdens.
Morgan, 1990 [22]	C	Described environmental health practice as combating and controlling the causative agents of disease before humans are exposed, reducing human suffering and death. Morgan further details that it encompasses complex relationships between environments in which people live and their state of health.
LaFollette et al., 1999 [23]	C	Characterised the traditional regulatory paradigm of environmental health practice to comprise: inspection, compliance, re-inspection and enforcement procedures. La Follette and colleagues then describe an emergent paradigm for environmental health practice that they name the participatory education paradigm. In contrast to the traditional, this includes: needs and capacity assessment, program planning, program implementation, and evaluation.
Parkes et al., 2003 [24]	C	Adopted a more critical view of environmental health practitioners, concerning themselves with predominantly biophysical effects of the environment on human health, and while greater awareness of human behaviour and social processes was identified to be emerging amongst practitioners, the basic approach to environmental health practice remained largely focused on preventing hazardous environmental exposures.
Powis et al., 2003 [25]	CS	When describing transformations of practice and policy in environmental health, Powis and colleagues noted a reorientation of traditional focus of practice on health protection toward integration of health promotion conventions, particularly with respect to the creation of ‘healthy settings’.
Howze et al., 2004 [26]	C	Described traditional environmental health practice to largely comprise risk assessment. In doing so, Howze and colleagues explain risk assessment to comprise four major components: hazard identification, dose–response assessment, exposure assessment, and risk characterisation. They further describe that where a significant risk is determined to be present, then a risk management response may be employed that may include one or more of the following strategies: source control, path control, control at the person level, and secondary prevention.
Choo et al., 2012 [8]	RF	Noted environmental health practitioners must possess a wide range of competencies to be effective at promoting and improving environmental health. In addition to technical skills, competencies in assessment, management and communication were identified as necessary to support effective environmental health practice.
Reynolds & Wills, 2012 [27]	R	Described environmental health practice to be concerned with protecting health from physical, social, chemical or biological aspects of the environment. The major approach of practice to exerting this ‘protection’ is through enforcement actions, and while more contemporary conceptualisations of practice include elements of health promotion and improvement of health and wellbeing, many practitioners engaged in practice felt limited capacity to step beyond the traditional enforcement realm.
Rideout et al., 2016 [28]	C	In an exploration of the capacity for environmental health practitioners to promote health through facilitating healthy settings in built environments, Rideout and colleagues reflected on traditional environmental health practice as safeguarding health through control of immediate hazards and infectious disease agents within the environment. Rideout and colleagues instead implore practitioners to adopt a broader view to practice that protects people from environments that do not support health.
Brooks et al., 2019 [29]	C	Described the responsibility of environmental health practitioners to address environment-related threats and determinants of health. Identifying that environmental health practice is the delivery of services routinely to prevent adverse health outcomes and plan, respond and recover from biological, natural and anthropogenic disasters. They noted that environmental health practitioners are strategically positioned to diagnose, intervene, and prevent public health threats.
Musoke et al., 2021 [30]	C	Described the role of environmental health practitioners to have a focus on preventive health, adopting a risk-based lens in the assessment and control of physical, chemical and biological environmental factors that are likely to affect human health. Special note was made of preventing the spread of infectious disease and its complement to the control of antimicrobial resistance.
Oerther 2021 [31]	C	Adopted a very progress-focused view when suggesting the conceptual framework that underpins environmental health practice recognises good health as a normal state of the public, and thus the aim of practice is to prevent deviation from that normal state, or promote wellbeing through environmental improvements.
Rodrigues et al., 2021 [32]	R	Viewing environmental health practice in times of public health emergency, Rodrigues and colleagues identified the values characterising environmental health practice to include building hazard resilience and community capacity to respond and recover from an emergency, hazard identification and risk assessment, enhancing routine health protection services, and recontextualising approaches to health protection within the traditional domains of environmental health practice to a disaster context.
Ross et al., 2024 [33]	R	Defined environmental health as aspects of human health determined by physical, chemical, biological, social and psychosocial factors in the environment and thus, described environmental health practice as the regulation of these factors with the use of regulatory tools devised and applied to oversee safety in commercial settings where risk of hazardous exposures is elevated. They further noted environmental health practice to involve conducting risk assessment, health impact assessment, public health planning, disaster response, and health protection within broader contexts.

C—Commentary, CS—Case Study, R—Research, RF—Reflection.

**Table 2:** Applying the narrative to domains of environmental health practice.

Domain of Environmental Health Practice	Means of Inquiry	Duty Holder	Focus of Assessment	Environmental Health Hazards	Interventions Applied
Food safety	Inspection, investigation, sampling, laboratory analysis, measurement	Food business, Food business operator	Elimination or control of hazards by duty holder	Chemical, physical, biological	Education, advice, seizure, directions, orders, prohibition, penalties, prosecution
Water quality	Inspection, investigation, sampling, laboratory analysis, measurement	Water suppliers, Aquatic facility operators, Land managers	Elimination or control of hazards by duty holder	Chemical, physical, biological	Education, advice, directions and orders, prohibition, penalties, prosecution
Wastewater management	Inspection, investigation, sampling, laboratory analysis, measurement	Land owners, Water authorities, Waste generators	Elimination or control of hazards by duty holder	Chemical, physical, biological	Education, advice, directions, orders, prohibition, penalties, prosecution
Statutory nuisance	Investigation, sampling, laboratory analysis, measurement	Emitters, Generators	Elimination or control of hazards by duty holder	Chemical, physical, biological	Education, advice, directions, orders, prohibition, penalties, prosecution
Infection control	Inspection, investigation, sampling, laboratory analysis, measurement	Personal service operators, Institutional setting operators	Elimination or control of hazards by duty holder	Biological	Education, advice, directions, orders, prohibition, penalties, prosecution
Housing standards	Inspection, investigation, sampling, laboratory analysis, measurement	Property owners, Property managers, Housing providers	Elimination or control of hazards by duty holder	Chemical, physical, biological	Education, advice, directions, orders, prohibition, penalties, prosecution
Built environment	Inspection, investigation, health impact assessment, sampling, laboratory analysis, measurement	Developers, Land owners, Land managers	Elimination or control of hazards by duty holder	Chemical, physical, biological	Education, advice, directions, orders, prohibition, penalties, prosecution
Site contamination	Inspection, investigation, sampling, laboratory analysis, measurement	Land owners, Land managers	Elimination or control of hazards by duty holder	Chemical	Education, advice, directions, orders, prohibition, penalties, prosecution
Pollution control	Inspection, investigation, sampling, laboratory analysis, measurement	Emitters, Generators, Waste service operators	Elimination or control of hazards by duty holder	Chemical, physical, biological	Education, advice, directions, orders, prohibition, penalties, prosecution
Vector control	Inspection, investigation, sampling, laboratory analysis, measurement	Land owners, Land managers	Elimination or control of hazards by duty holder	Biological	Education, advice, directions, orders, prohibition, penalties, prosecution
Microplastics *	Sampling, laboratory analysis, measurement	Water suppliers, Food producers, Manufacturers, Distributors, Emitters, Generators	Elimination or control of hazards by duty holder	Chemical, physical	Education, advice, directions, orders, prohibition, penalties, prosecution
PFAS *	Sampling, laboratory analysis, measurement	Water suppliers, Waste managers, Land managers, Manufacturers, Distributors, Emitters, Generators	Elimination or control of hazards by duty holder	Chemical	Education, advice, directions, orders, prohibition, penalties, prosecution
Antimicrobial resistance *	Inspection, investigation, sampling, laboratory analysis, measurement, surveillance	Food producers, Wastewater managers, Healthcare providers, Manufacturers	Elimination or control of hazards by duty holder	Biological	Education, advice, directions, orders, prohibition, penalties, prosecution

* Emerging environmental health issues.

**Table 3:** Strategies for building brand equity for environmental health practice.

Brand equity	Apply a coordinated effort in communication and expression of the brand. Maintain consistent representation of the brand and the central narrative. Ensure alignment of sub-brands to the overarching brand of environmental health practice.
Brand relevance	4. Share our narrative and our unique value proposition at every opportunity, as individuals and as a collective, to build visibility. 5. Build credibility by recognising the importance of an accurate and consistent central narrative to ensure the work we do and the services we deliver are what we say we do.
Brand image	6. Actively share the purpose and narrative of our brand with the beneficiaries of our work to assist in establishing differentiation. 7. Demonstrate application of scientific, authoritative, evidence-informed, risk-focused approaches in the work we do. 8. Advocate for appropriate tools and resources required for our work to ensure we are equipped to deliver quality services and results.
Brand loyalty	9. Partner with policy makers to establish or maintain our role and responsibilities in statute and health protection systems. 10. Support policy makers to identify and assign emerging roles and responsibilities aligning with our remit appropriately to our discipline.
Brand portfolio	11. Build a brand portfolio comprising sub-brands that include sub-disciplines, peak bodies and geographical chapters of environmental health practice.

## Data Availability

The original contributions presented in this study are included in the article. Further inquiries can be directed to the corresponding author.

## Abbreviations

The following abbreviations are used in this manuscript:

COVID-19	Coronavirus Disease 2019
EBSCO	Elton B. Stephens Company
CINAHL	Cumulative Index to Nursing and Allied Health Literature
EMBASE	Excerpta Medica Database
